# Interactive Affection of Pre-Pregnancy Overweight or Obesity, Excessive Gestational Weight Gain and Glucose Tolerance Test Characteristics on Adverse Pregnancy Outcomes Among Women With Gestational Diabetes Mellitus

**DOI:** 10.3389/fendo.2022.942271

**Published:** 2022-07-07

**Authors:** Li-hua Lin, Juan Lin, Jian-ying Yan

**Affiliations:** ^1^ Department of Healthcare, Fujian Maternity and Child Health Hospital, Fuzhou, China; ^2^ Department of Obstetrics, Fujian Maternity and Child Health Hospital, Fuzhou, China

**Keywords:** adverse pregnancy outcomes, overweight or obesity, excessive gestational weight gain, gestational diabetes mellitus, multiplicative interaction

## Abstract

**Purpose:**

To examine the combined effect of pre-pregnancy overweight or obesity, excessive gestational weight gain, and glucose tolerance status on the incidence of adverse pregnancy outcomes among women with gestational diabetes mellitus.

**Methods:**

A observational study including 5529 gestational diabetes mellitus patients was performed. Logistic regression were used to assess the independent and multiplicative interactions of overweight or obese, excessive gestational weight gain, abnormal items of oral glucose tolerance test and adverse pregnancy outcomes. Additive interactions were calculated using an Excel sheet developed by Anderson to calculate relative excess risk.

**Results:**

Overall 1076(19.46%) study subject were overweight or obese and 1858(33.60%) women gained weight above recommended. Based on IADPSG criteria, more than one-third women with two, or three abnormal glucose values. Preconception overweight or obesity, above recommended gestational weight gain, and two or more abnormal items of oral glucose tolerance test parameters significantly increased the risk of adverse pregnancy outcomes, separately. After accounting for confounders, each two of overweight or obesity, excessive gestational weight gain, two or more abnormal items of OGTT parameters, the pairwise interactions on adverse pregnancy outcomes appear to be multiplicative. Coexistence of preconception overweight or obesity, above recommended gestational weight gain and two or more abnormal items of oral glucose tolerance test parameters increased the highest risk for adverse pregnancy outcomes. No additive interaction was found.

**Conclusions:**

Pre-pregnancy overweight or obesity, excessive gestational weight gain, two or more abnormal items of OGTT parameters contribute to adverse pregnancy outcomes independently among women with gestational diabetes mellitus. Additionally, the combined effect between these three factors and adverse pregnancy outcomes appear to be multiplicative. Interventions focus on maternal overweight or obesity and gestational weight gain should be offered to improve pregnancy outcomes.

## Introduction

Gestational diabetes mellitus(GDM) is one of the common complications during pregnancy (1). In the past decades, the prevalence of gestational diabetes mellitus(GDM) has increased rapidly and caused a tremendous disease burden (2, 3). In China, the prevalence is continue climbing due to the growing number of childbearing age women, the overweight or obesity epidemic, correlated to the implementation of the “two-child policy” since October 2015 (1). The risk factor includes older age, family history of diabetes, pre-pregnancy overweight or obesity, previous GDM, excessive gestational weight gain, polycystic ovarian syndrome(PCOS) (1, 4–7). In China, the GDM has become epidemic and affects the short- and long-term health of mothers and their offspring such as hypertensive disorders of pregnancy(HDP), preeclampsia, cesarean section, shoulder dystocia for mother and large gestational age, premature, macrosomia and even increase the risks of obesity, type 2 diabetes mellitus(T2DM), and metabolic syndrome in adult life (8–15). overweight or obesity have increased rapidly across different age groups and sexes including childbearing age women which not only increase the rates of metabolic complication but also contribute to greater risks of adverse pregnancy outcomes (3, 16). Gestational weight gain is strongly associated with maternal and fetal growth health. The Institute of Medicine(IOM) introduced guidelines for gestational weight gain to lower the risks of perinatal complications in 1990 and updated in 2009 (17). Extensive research has shown the increased risk for maternal and infant adverse pregnancy outcomes with excessive gestational weight gain including HDP, GDM, large gestation age for newborns, cesarean section, macrosomia, and childhood obesity (17–19). Moreover, it has been demonstrated that there was a significantly increased risk of cesarean section, preterm delivery, macrosomia and large gestation age as the number of abnormal terms of oral glucose tolerance test(OGTT) increased (20).

On the face of these, women with GDM, overweight or obesity, and excessive gestational weight gain are all independent associated with increased risk of adverse maternal and perinatal outcomes. When co-occurrence of these three factors, maternal with overweight or obesity and excessive gestational weight gain may increase their already elevated risk of adverse maternal and perinatal outcomes, particularly in GDM women with two or three abnormal terms of glucose values on the OGTT. However, in women with GDM, the combination effect of pre-pregnancy overweight or obesity, excessive gestational weight gain and glucose tolerance status for adverse maternal and perinatal outcomes is still unknown. Hence, this study aims to evaluate the combined effect of pre-pregnancy overweight or obesity, excessive gestational weight gain, and glucose tolerance status on pregnancy outcomes among women with GDM.

## Materials and Methods

### The Study Population

The observational study reviewed medical records in Fujian Maternity and Child Health Hospital of women who were diagnosed with GDM and delivery of a live singleton neonate after 28 weeks gestation between 2017 and 2021. The eligibility criteria include all women who received perinatal care and performed a 75 g OGTT between 24 and 28 weeks of gestation. We excluded those with preconception diabetes mellitus and incomplete medical records.

This was a retrospective study and was approved by the Ethics Committee of Fujian Maternity and Child Health Hospital.

### Study Variables

All the medical record data were extracted into a computerized database including demographic data, obstetric data, and delivery data including age, pre-pregnancy weight, height, gravity, parity, maternal weight at each perinatal examination, and OGTT values, discharge diagnosis, gestational age at delivery and neonatal data.

pre-pregnancy body mass index(BMI) was calculated as [pre-pregnancy weight(kg)/height^2^(m^2^)] based on self-reported pre-pregnancy and measured height in hospital. pre-pregnancy BMI was classified as underweight and normal weight (BMI<24.0kg/m^2^), overweight or obesity (BMI≥24.0kg/m^2^) based on Chinese standard (21). Gestational weight gain was calculated as the difference between pre-pregnancy weight and delivery weight. The gestational weight gain was divided as above recommendations and as or below recommendations according to weight monitoring and evaluation during the pregnancy period of Chinese women (Underweight, >16.0kg; Normal weight,>14.0kg; Overweight,>11.0kg; Obesity,>9.0kg) (22). We used the OGTT result to classify the GDM group into one abnormal item and at least two abnormal items of OGTT parameters: fasting ≥5.10 mmol/L,1 h ≥10.0 mmol/L, or 2 h ≥8.5 mmol/L (23).

The main pregnancy outcomes in this study included: macrosomia, defined as a birth weight of more than 4000g (24); Large for gestational age (LGA) or small for gestational age(SGA), defined as a birth weight more than 90^th^ or less than 10^th^ percentile based on gender and gestational age (25); Preterm delivery, defined as gestational age at delivery<37 weeks but >28 weeks; Full term low birth weight, defined as a gestational age ≥ 37weeks with birth weight less than 2500g; hypertensive disorders of pregnancy(HDP), defined as blood pressure ≥140/90 mmHg that occurred after 20 weeks gestation but without proteinuria (26). Other pregnancy outcomes were cesarean section and composite outcome. Composite outcome was defined as either one of macrosomia, LGA, SGA, preterm delivery, full term low birth weight, HDP, and cesarean section.

### Statistical Analysis

Data of maternal demographic and obstetrical and neonatal outcomes were tested for normal distribution and shown as median (inter-quartile range, IQR) for continuous variables as the abnormal distribution, number (percentage) for categorical variables.

Binary logistic regression was used to analyze the effect of overweight or obesity/excessive gestational weight gain/at least two abnormal items of OGTT parameters on the pregnancy outcomes and multiplicative interactions, expressed resulted as odds ratio(OR) and 95% confidence intervals(95%CI). For all outcomes except preterm delivery, the adjustment OR was based on the maternal age, gestational age, infant sex, gravity, and parity. For preterm delivery, the adjustment OR was based on the maternal age, infant sex, gravity, and parity. Multiplicative interactions among individual risk factor was carried out by adding two or more product terms to the regression model while statistical significance indicates the multiplicative interactions. Additive interactions were calculated using an Excel sheet prepared by Anderson to calculate the following three indices with their 95% CIs: relative excess risk due to interaction (RERI), attributable proportion due to interaction (AP), and interaction index (synergy index, SI) (27). The 95% CI of RERI and AP include “0” and the 95% CI of SI include “1” indicate that there is no summation interaction. We firstly performed a logistic regression model to calculate the regression coefficients and covariance matrix for each two of factors and then they were input the Excel sheet to calculate RERI, AP, SI and their 95% CIs. Statistical analysis was performed using IBM SPSS Statistics 22. 0 software.

## Results

### Basic Characteristics of the Participants

A total of 8850 pregnant women with GDM received prenatal examinations and delivery at Fujian Provincial Maternity and Children’s Hospital between 2017 and 2021. We excluded 3321women from the following: 58 pregnant women who terminated the pregnancy before 28 weeks, 212 women with multiple pregnancies, 18 women with stillbirth, 340 women without gestational weight value before delivery, and 2693 women who didn’t undergo a complete oral glucose tolerance test. Of the remained 5529 GDM women, the median maternal age was 31 years, pre-pregnancy weight was 54kg;12.41% were underweight, 68.31% were normal weight, 18.09% were overweight, and 1.37%were obese. Overall, more one-third of women were of greater gravidity, more than 3 times. Of the 5529 GDM women, 47.98% were multiparity. According to the Chinese gestational weight gain guidelines (22), 14.74% of women gained weight below recommended, 51.65% f women gained weight as recommended and 33.60% of women gained weight above recommended. As for glucose tolerance status, our study group consisted of 59.54%, 31.33%, and 9.13% women with one, two, or three abnormal glucose values, respectively. More than 37% of women are delivered by cesarean section. The characteristics of the mothers and neonates are described in **Table 1**. Due to the small number of obese GDM women, we merged overweight and obese GDM women into the same group and analyzed these parameters as just one variable.

**Table 1:** Basic characteristics of the participants (n = 5529).

Variables	All participants
Maternal age (median[IQR],Years)	31[28,35]
Pre-pregnancy Weight (median[IQR],Kg)	54[49.5,60]
**Body mass index category (N,%)**	
Underweight	686 (12.41)
Normal weight	3767 (68.13)
Overweight	1000(18.09)
Obesity	76 (1.37)
**Gravidity**	
1	1972 (35.67)
2	1679 (30.37)
≥3	1878 (33.97)
**Parity (N,%)**	
Primiparity	2876 (52.02)
Multiparity	2653 (47.98)
**Gestational age at delivery (median[IQR],Weeks)**	39[38,40]
**Gestational weight gain category by Chinese guideline (N,%)**	
Below recommended	815 (14.74)
As recommended	2856 (51.65)
Above recommended	1858 (33.60)
**Birth weight (median[IQR],g)**	3300[3032.5,3570]
**Glucose tolerance status (N,%)**	
One abnormal item	3292 (59.54)
Two abnormal items	1732 (31.33)
Three abnormal items	505 (9.13)
**Model of delivery (N,%)**	
Vaginal birth	3463 (62.63)
Cesarean delivery	2066 (37.37)
**Infant sex (N,%)**	
Boy	2981 (53.92)
Girl	2548 (46.08)

### Association of Overweight or Obesity, Excessive Gestational Weight Gain, Abnormal Items of OGTT With Adverse Pregnancy Outcomes

The association between overweight or obesity, excessive gestational weight gain, two or more abnormal items of OGTT parameters, and adverse pregnancy outcomes were analyzed by binary logistic regression and is shown in **Table 2**. Women with overweight or obesity had an increased relative risk of macrosomia (aOR:1.83,95%CI:1.39~2.40), LGA(aOR:1.59,95%CI:1.36~1.85), HDP(aOR:2.73,95%CI:2.15~3.46), cesarean section(aOR:1.58,95%CI:1.38~1.82) and composite outcome(aOR:1.82,95%CI:1.57~2.10). Compared to women with gestational weight gain follow or below recommendations, women with gestational weight gain above recommendations was associated with an increased risk of macrosomia(aOR:2.24,95%CI:1.75~2.88), LGA(aOR:1.73,95%CI:1.52~1.98), HDP(OR:1.87,95%CI:1.49~2.36), cesarean section(aOR:1.49,95%CI:1.32~1.68) and composite outcome(aOR:1.59,95%CI:1.41~1.79), but decreased the risk of preterm delivery(aOR:0.76,95%CI:0.59~0.99). There also have an association between at least two abnormal items of OGTT parameters and perinatal outcomes. The aOR of the association for macrosomia was 1.31(95%CI:1.02~1.68), for LGA was 1.16 (95%CI:1.02~1.32), for HDP was 1.48(95%CI:1.18~1.85).

**Table 2:** Association of overweight or obesity, excessive gestational weight gain and glucose tolerance status with adverse pregnancy outcomes^a^.

Category	Macrosomia	LGA	Preterm delivery^b^	HDP	SGA	Cesareansection	Full term low birth weight	Composite outcome
**Pre-pregnancy BMI category (kg/m^2^)**								
Underweight or Normal(<24.0)	1.00 (Ref.)	1.00 (Ref.)	1.00 (Ref.)	1.00(Ref.)	1.00 (Ref.)	1.00 (Ref.)	1.00 (Ref.)	1.00 (Ref.)
Overweight or Obese(≥24.0)	1.83 (1.39,2.40)	1.59 (1.36,1.85)	0.95 (0.70,1.29)	2.73 (2.15,3.46)	0.80 (0.49,1.31)	1.58 (1.38,1.82)	0.72 (0.37,1.40)	1.82 (1.57,2.10)
**Gestational weight gain category**								
As or below recommended	1.00 (Ref.)	1.00 (Ref.)	1.00 (Ref.)	1.00 (Ref.)	1.00 (Ref.)	1.00 (Ref.)	1.00 (Ref.)	1.00 (Ref.)
Above recommended	2.24 (1.75,2.88)	1.73 (1.52,1.98)	0.76 (0.59,0.99)	1.87 (1.49,2.36)	0.70(0.65,1.04)	1.49 (1.32,1.68)	0.64 (0.36,1.16)	1.59 (1.41,1.79)
**Glucose tolerance status**								
One abnormal item	1.00 (Ref.)	1.00 (Ref.)	1.00 (Ref.)	1.00 (Ref.)	1.00 (Ref.)	1.00 (Ref.)	1.00 (Ref.)	1.00 (Ref.)
At least two abnormal items	1.31 (1.02,1.68)	1.16 (1.02,1.32)	1.26 (0.99,1.60)	1.48 (1.18,1.85)	0.95 (0.66,1.37)	1.04 (0.93,1.17)	0.84 (0.50,1.38)	1.09 (0.97,1.22)

Data are showed as OR(95%CI).

BMI, body mass index; LGA, large for gestational age; SGA, small for gestational age; HDP, Hypertensive disorders of pregnancy; ^a^ For all outcomes except preterm delivery were adjusted for maternal age, gestational age, infant sex, gravity, and parity.^b^ adjusted for on the maternal age, infant sex, gravity, and parity.

### Pairwise Interaction of Overweight or Obesity, Excessive Gestational Weight Gain and Abnormal Items of OGTT on Adverse Pregnancy Outcomes

Pairwise interaction analysis showed the multiplicative interactions were observed between overweight or obesity with gestational weight gain above recommendations and macrosomia(aOR:3.58,95%CI:2.48~5.16), LGA(aOR:2.67,95%CI:2.15~3.31), HDP(aOR:3.97,95%CI:2.84,5.54), cesarean section(aOR:2.27,95%CI:1.85~2.78) and composite outcome(aOR:2.97,95%CI:2.36~3.73). In addition, there was a positive multiplicative interactions between overweight or obesity with at least two abnormal items of OGTT parameters and macrosomia(aOR:2.05,95%CI:1.39~3.04), LGA(aOR:1.72,95%CI:1.39~2.12),HDP(aOR:3.72,95%CI:2.69,5.12),cesarean section(aOR:1.59,95%CI:1.30~1.93) and composite outcome(aOR:1.94, 95%CI:1.57~2.39). Similar multiplicative interactions of gestational weight gain with two or more abnormal items of OGTT parameters and macrosomia (aOR:3.17,95%CI:2.19~4.58), HDP(aOR:2.90,95%CI:2.06~4.09), cesarean section(aOR:1.57,95%CI:1.31~1.89) and composite outcome(aOR:1.77, 95%CI:1.46~2.13) (**Table 3**).

**Table 3:** Pairwise interaction of overweight or obesity, excessive gestational weight gain and glucose tolerance status on adverse pregnancy outcomes^a^.

Category		Macrosomia	LGA	Preterm delivery^b^	HDP	SGA	Cesarean section	Full term low birth weight	Composite outcome
**Pre-pregnancy BMI category(kg/m)**	**Gestational weight gain category**								
Underweight or Normal(<24.0)	As or below recommended	1.00(Ref.)	1.00(Ref.)	1.00(Ref.)	1.00(Ref.)	1.00(Ref.)	1.00(Ref.)	1.00(Ref.)	1.00(Ref.)
	Above recommended	2.15 (1.60,2.89)	1.58 (1.36,1.84)	0.63 (0.46,0.87)	134 (1.44,2.57)	1.03 (0.58,1.81)	1.39 (1.21,1.60)	0.81 (0.43,1.54)	1.44 (1.26,1.65)
Overweight or Obese(≥24.0)	As or below recommended	1.68 (1.12,2.52)	1.35 (1.10,1.66)	0.72 (0.48,1.09)	3.03 (2.19,4.19)	0.79 (0.51,1.22)	1.43 (1.20,1.72)	1.01 (0.48,2.13)	1.55 (1.29,1.87)
	Above recommended	3.58 (2.48, 5.16)	2.67 (2.15,3.31)	1.02 (0.68,1.54)	3.97 (2.84,5.54)	0.42 (0.17,1.05)	2.267 (1.85,2.78)	0.276 (0.07,1.17)	2.970 (2.36,3.73)
**Pre-pregnancy BMI category(kg/m^2^)**	**Glucose tolerance status**								
Underweight or Normal(<24.0)	One abnormal item	1.00(Ref.)	1.00(Ref.)	1.00(Ref.)	1.00(Ref.)	1.00(Ref.)	1.00(Ref.)	1.00(Ref.)	1.00(Ref.)
	At least two abnormal items	1.43 (1.07,1.92)	1.16 (0.99,1.34)	1.17 (0.90,1.53)	1.34 (1.00,1.78)	0.95 (0.64,1.42)	1.02 (0.90,1.17)	1.02 (0.59,1.76)	1.05 (0.93,1.20)
Overweight or Obese(≥24.0)	One abnormal item	2.20 (1.52,3.19)	1.66 (1.34,2.04)	0.75 (0.47,1.20)	2.52 (1.78,3.57)	0.78 (0.40,1.54)	1.60 (1.33,1.94)	1.14 (0.51,2.53)	1.78 (1.46,2.17)
	At least two abnormal items	2.05 (1.39,3.04)	1.72 (1.39,2.12)	1.31 (0.89,1.94)	3.72 (2.69,5.12)	0.80 (0.40,1.57)	1.59 (1.30,1.93)	0.36 (0.11,1.22)	1.94 (1.57,2.39)
**Gestational weight gain category**	**Glucose tolerance status**								
As or below recommended	One abnormal item	1.00(Ref.)	1.00(Ref.)	1.00(Ref.)	1.00(Ref.)	1.00(Ref.)	1.00(Ref.)	1.00(Ref.)	1.00(Ref.)
	At least two abnormal items	1.41 (0.99,2.00)	1.19 (1.01,1.40)	1.19 (0.90,1.57)	1.64 (1.21,2.23)	0.93 (0.61, 1.41)	1.08 (0.94, 1.25)	1.01 (0.58, 1.77)	1.13 (0.99, 1.30)
Above recommended	One abnormal item	2.33 (1.66,3.26)	1.74 (1.47,2.07)	0.72 (0.50,1.02)	2.08 (1.50,2.87)	0.69 (0.42, 1.14)	1.53 (1.31, 1.78)	0.89 (0.44, 1.77)	1.63 (1.40, 1.89)
	At least two abnormal items	3.17 (2.19,4.58)	2.09 (1.71,2.55)	1.01 (0.69,1.49)	2.90 (2.06, 4.09)	0.65 (0.35, 1.23)	1.57 (1.31, 1.89)	0.31 (0.09, 1.04)	1.77 (1.46, 2.13)

Data are showed as OR(95%CI).

BMI, body mass index; LGA, large for gestational age; SGA, small for gestational age; HDP, Hypertensive disorders of pregnancy; ^a^ For all outcomes except preterm delivery were adjusted for maternal age, gestational age, infant sex, gravity, and parity. ^b^ adjusted for on the maternal age, infant sex, gravity, and parity.

When each two of overweight or obesity, excessive gestational weight gain, two or more abnormal items of OGTT parameters exist at the same time, no additive interaction was found for adverse pregnancy outcomes. However, neither multiplication interaction nor additive interaction was noticed in preterm delivery, SGA, and full-term low birth weight (**Tables 3** and **4**).

**Table 4:** Additive interaction of overweight or obesity, excessive gestational weight gain and glucose tolerance status on adverse pregnancy outcomes.

	Iterm1	Iterm2	Additive interaction		
			RERI (95%CI)	AP (95%CI)	SI (95%CI)
**Macrosomia**	Pre-pregnancy overweight or obese(≥24.0)	Gestational weight gain above recommendations	0.58(-2.47,3.63)	0.171(-0.50,0.84)	1.32(0.45,3.88)
	Pre-pregnancy overweight or obese(≥24.0)	At least two abnormal items of Glucose tolerance	-0.86(-1.86,0.69)	-0.29(-1.30,0.73)	0.64(0.12,3.57)
	Gestational weight gain above recommendations	At least two abnormal items of Glucose tolerance	0.44(-2.30,3.17)	0.14(-0.55,0.82)	1.25(0.42,3.75)
**LGA**	Pre-pregnancy overweight or obese(≥24.0)	Gestational weight gain above recommended	0.74(-1.27,2.76)	0.28(-0.14,0.67)	1.80(1.043.097)
	Pre-pregnancy overweight or Obese(≥24.0)	At least two abnormal items of Glucose tolerance	-1.57(-1.94,-1.20)	-12.20(-39.68,15.28)	0.88(0.19,4.06)
	Gestational weight gain above recommendations	At least two abnormal items of Glucose tolerance	-0.10(-1.03,0.84)	-0.06(-0.68,0.56)	0.88(0.19,4.06)
**Preterm delivery**	Pre-pregnancy body mass index Overweight or Obese(≥24.0)	Gestational weight gain above recommendations	0.66(-0.22,1.54)	NA	NA
	Pre-pregnancy body mass index Overweight or Obese(≥24.0)	At least two abnormal items of Glucose tolerance	0.39(-0.73,1.52)	0.299(-0.13,0.73)	NA
	Gestational weight gain above recommendations	At least two abnormal items of Glucose tolerance	-0.17(-0.44,0.10)	-3.39(-6.21,-0.58)	1.22(0.85,1.75)
**HDP**	Pre-pregnancy body mass index Overweight or Obese(≥24.0)	Gestational weight gain above recommendations	0.02(-3.09,3.13)	0.01(-0.77,0.78)	1.01(0.35,2.86)
	Pre-pregnancy body mass index Overweight or Obese(≥24.0)	At least two abnormal items of Glucose tolerance	0.87(-2.38,4.11)	0.23(-0.34,0.81)	1.47(0.58,3.69)
	Gestational weight gain above recommendations	At least two abnormal items of Glucose tolerance	0.87(-2.47,4.20)	0.23(-0.37,0.83)	1.47(0.55,3.91)
**SGA**	Pre-pregnancy body mass index Overweight or Obese(≥24.0)	Gestational weight gain above recommendations	-0.75(-1.34,2.81)	0.48(-0.08,1.04)	2.62(0.25,27.69)
	Pre-pregnancy body mass index Overweight or Obese(≥24.0)	At least two abnormal items of Glucose tolerance	0.06(-0.68,081)	0.08(-0.75,0.90)	0.77(0.01,49.04)
	Gestational weight gain above recommendations	At least two abnormal items of Glucose tolerance	0.04(-0.40,0.48)	0.06(-0.51,0.63)	0.90(0.21,3.80)
**Cesarean section**	Pre-pregnancy body mass index Overweight or Obese(≥24.0)	Gestational weight gain above recommendations	0.44(-1.11,1.99)	0.19(-0.25,0.64)	1.53(0.82,2.88)
	Pre-pregnancy body mass index Overweight or Obese(≥24.0)	At least two abnormal items of Glucose tolerance	-0.04(-0.80,0.71)	-0.03(-0.54,0.49)	0.93(0.22,4.00)
	Gestational weight gain above recommendations	At least two abnormal items of Glucose tolerance	-0.04(-0.82,0.75)	-0.03(-0.56,0.51)	0.94(0.21,4.34)
**Full term low birth weight**	Pre-pregnancy body mass index Overweight or Obese(≥24.0)	Gestational weight gain above recommendations	-0.55(-1.27,0.18)	-1.97(-6.90,2.96)	4.04(0.03,544.94)
	Pre-pregnancy body mass index Overweight or Obese(≥24.0)	At least two abnormal items of Glucose tolerance	-0.79(-1.56,-0.03)	-2.18(-6.89,2.53)	-4.03(NA)
	Gestational weight gain above recommendations	At least two abnormal items of Glucose tolerance	-0.59(-1.06,-0.11)	-1.89(-5.88,2.10)	6.53(0.01,20.15)
**Composite outcome**	Pre-pregnancy body mass index Overweight or Obese(≥24.0)	Gestational weight gain above recommended	0.98(-1.39,3.35)	0.33(-0.07,0.73)	1.99(1.28,3.10)
	Pre-pregnancy body mass index Overweight or Obese(≥24.0)	At least two abnormal items of Glucose tolerance	0.10(-1.03,1.23)	0.05(-0.46,0.57)	1.12(0.41,3.07)
	Gestational weight gain above recommendations	At least two abnormal items of Glucose tolerance	0.01(-0.97,0.98)	0.004(-0.54,0.55)	1.01(0.29,3.55)

NA, Not Applicable; LGA, large for gestational age; SGA, small for gestational age; HDP, Hypertensive disorders of pregnancy; RERI, relative excess risk; AP, attributable proportion; SI, synergy index.

### Coexist Interaction of Overweight or Obesity, Excessive Gestational Weight Gain and Abnormal Items of OGTT on Adverse Pregnancy Outcomes

Compared to underweight or normal, gestational weight gain as or blew recommendations and with only one abnormal term of OGTT pathological values pregnant women, women with overweight or obesity, gestational weight gain above recommendations and with two or more abnormal items of OGTT parameters had highest risks of macrosomia(aOR:4.52,95%CI:2.60~7.84), LGA(aOR:3.19,95%CI:2.33~4.37), HDP(aOR:7.60, 95%CI:4.78~12.08), cesarean section(aOR:2.56, 95%CI:1.89,3.47) and composite outcome(aOR:3.75, 95%CI:2.63,5.34), after controlling the confounding factors.

Furthermore, gestational weight gain as or below recommendations among overweight or obesity women also increased relative risk of macrosomia[(aOR:2.12, 95%CI:1.19,3.78) and (aOR:2.01, 95%CI:1.13,3.73)], LGA[(aOR:1.50, 95%CI:1.12,2.02) and (aOR:1.44, 95%CI:1.12,1.94)], HDP[(aOR:3.91, 95%CI:2.29,5.96) and (aOR:4.11, 95%CI:2.41,6.34)], cesarean section[(aOR:1.55, 95%CI:1.19,2.00) and (aOR:1.42, 95%CI:1.10,1.83)], and composite outcome[(aOR:1.61, 95%CI:1.24,2.09) and (aOR:1.67, 95%CI:1.28,2.15)], regardless of OGTT pathological values. Moreover, gestational weight gain as or below recommendations among underweight or normal women with two or more abnormal items of OGTT parameters was associated with increased risk of macrosomia(aOR:1.52, 95%CI:1.01,2.29), LGA(aOR:1.21, 95%CI:1.01,1.46), HDP(aOR:1.84, 95%CI:1.26,2.70), but do not effect on cesarean section(aOR:1.10, 95%CI:0.94,1.28) and composite outcome(aOR:1.13, 95%CI:0.98,1.32) (**Table 5**).

**Table 5:** Coexist interaction of overweight or obesity, excessive gestational weight gain and glucose tolerance status on adverse pregnancy outcomes^a^.

Pre-pregnancy BMI category(kg/m^2^)	Gestational weight gain category	Glucose tolerance status	Macrosomia	LGA	Preterm delivery^b^	HDP	SGA	Cesarean section	Full term low birth weight	Composite outcome
Underweight or Normal (<24.0)	As or below recommended	One abnormal items	1.00(Ref.)	1.00 (Ref.)	1.00 (Ref.)	1.00 (Ref.)	1.00 (Ref.)	1.00 (Ref.)	1.00 (Ref.)	1.00 (Ref.)
		At least two abnormal items	1.52 (1.01,2.29)	1.21 (1.01,1.46)	1.13 (0.83,1.52)	1.84 (1.26,2.70)	0.97 (0.61,1.54)	1.10 (0.94,1.28)	1.24 (0.67,2.29)	1.13 (0.98,1.32)
	Above recommended	One abnormal items	2.22 (1.49,3.32)	1.63 (1.34,1.98)	0.62 (0.42,0.93)	2.58 (1.74,3.82)	0.85 (0.50,1.44)	1.49 (1.25,1.78)	1.19 (0.56,2.54)	1.55 (1.34,1.84)
		At least two abnormal items	3.37 (2.15,5.26)	1.84 (1.44,2.34)	0.75 (0.46,1.22)	2.53 (1.57,4.08)	0.65 (0.30,1.39)	1.37 (1.10,1.71)	0.39 (0.09,1.68)	1.45 (1.17,1.80)
Overweight or Obese (≥24.0)	As or below recommended	One abnormal items	2.12 (1.19,3.78)	1.50 (1.12,2.02)	0.51 (0.26,1.03)	3.91 (2.29,5.96)	1.24 (0.60,2.60)	1.55 (1.19,2.00)	1.70 (0.69,4.23)	1.61 (1.24,2.09)
		At least two abnormal items	2.01 (1.13,3.73)	1.44 (1.12,1.94)	1.00 (0.60,1.67)	4.11 (2.41,6.34)	0.82 (0.34,1.95)	1.42 (1.10,1.83)	0.43 (0.10,1.89)	1.67 (1.28,2.15)
	Above recommended	One abnormal items	4.48 (2.71,7.4114)	2.7313 (2.040,3.66)	0.83 (0.45,1.53)	3.66 (2.17,6.15)	0.15 (0.02,1.12)	2.20 (1.67,2.90)	0.33 (0.04,2.54)	2.74 (2.04,3.68)
		At least two abnormal items	4.52 (2.60,7.84)	3.19 (2.33,4.37)	1.38 (0.80,2.38)	7.60 (4.78,12.08)	0.70 (0.25,1.99)	2.56 (1.89,3.47)	0.27 (0.35,2.11)	3.75 (2.63,5.34)

Data are showed as OR(95%CI).

BMI, body mass index; LGA, large for gestational age; SGA, small for gestational age; HDP: Hypertensive disorders of pregnancy; ^a^ For all outcomes except preterm delivery were adjusted for maternal age, gestational age, infant sex, gravity, and parity. ^b^ adjusted for on the maternal age, infant sex, gravity, and parity.

## Discussion

With the current epidemic of GDM, the public health of GDM is becoming more apparent in China. The incidence of GDM in China reached 8% (1), which is extremely harmful to maternal and child health. Therefore, the focus on the intervention of GDM is of great importance to reduce the risk of adverse perinatal outcomes. We found that pre-pregnancy overweight or obesity, above recommended gestational weight gain, and two or more abnormal items of OGTT parameters significantly increase the risk of adverse pregnancy outcomes, separately. After accounting for confounders, each two of overweight or obesity, excessive gestational weight gain, two or more abnormal items of OGTT parameters, the pairwise interactions on macrosomia, LGA, HDP, cesarean section, and the composite outcome appear to be multiplicative. Furthermore, coexistence of pre-pregnancy overweight or obesity, above recommended gestational weight gain and two or more abnormal items of OGTT parameters increase the highest risk for macrosomia, LGA, HDP, cesarean section, and composite outcome.

Overweight or obesity status before pregnancy in women of reproductive age influence not only the occurrence of GDM but also adverse perinatal outcomes. Growing studies has shown the higher pre-pregnancy BMI was an independent risk factor for LGA, HDP and cesarean section (28, 29), and when combined with GDM, acting as the most major determinant for macrosomia and LGA (30–32). This also accords with our finding, which also showed significant association with higher rate of HDP and composite outcome, after adjusting for the potential confounding variables.

Excessive gestational weight gain has been proved to be linked with adverse perinatal outcomes among GDM patients (33–35). We also found the similar results: exceeding the Chinese guideline (22) was linked with higher odds for macrosomia, LGA, HDP, cesarean section and composite outcome. Increased adipose tissue breakdown and circulating free fatty acids in third trimester may be transported through the placenta, promoting a pro-inflammatory environment with fetal metabolic programming consequences for the offspring (36–39). Alone or combined GDM and maternal obesity are independently associated with poor pregnancy outcomes. Maternal lipids may play a more important role in the fetal programming of infant obesity in the diabetic intrauterine environment (40). In the present study, we note that excessive gestational weight gain and overweight or obesity have higher risks on adverse pregnancy outcomes than maternal glucose characters. According to Pedersen’s hypothesis, elevated maternal glucose levels would increase fetal insulin production, leading to increased fetal growth and obesity (41). The result of HAPO showed excessive gestational weight gain was linked with increased fetal insulin production but no maternal glucose levels (42). Gestational weight gain may also affect fetal certain metabolic factors which can drive excess fetal growth. The HAPO study found than excess gestational weight gain increased fetal c-peptide (42). A prospective observational study conducted in Dublin found each 1kg increase in gestational weight gain was associated with a 0.039ng/ml increase in c-peptide and 0.024mmol/l decrease in cholesterol (40). Plasma c-peptide reflects the insulin secretory activity of pancreatic beta cells and the its higher level in cord blood was related to maternal insulin sensitivity, weight and adiposity maker (43–45).

Excessive gestational weight gain leads to increased insulin resistance and islet β-cell depletion, so that β-cells cannot secrete enough insulin to compensate for insulin resistance caused by pregnancy, leading to the occurrence of GDM. Adequate weight gain may be positive for perinatal outcomes among women with GDM. There is no consensus on the appropriate range of weight gain during pregnancy for GDM. Although Landon et al. observed parallel reductions of gestational weight gain and pre-eclampsia (46), reducing gestational weight gain has not been proven to reverse GDM-related complications (47). Thus, preventing excess gestational weight gain should be a important goal and feasible interventions for GDM.

Hyperglycemia is linked with adverse pregnancy outcomes. Maternal hyperglycemia allows for increased placental transfer of glucose and (fetal) beta-cell prohormones to the fetus, which leads to fetal hyperinsulinemia, causing fetal metabolic reprogramming that can lead to fetal overgrowth and/or obesity and so on (48). Feng et al (20) observed consistent trends between the number of abnormal OGTT parameters and odds of cesarean delivery, preterm delivery, and neonatal complications. Zhou et al (49) also found that the increasing number of abnormal OGTT parameters, the increased frequencies of LGA, and neonatal hypoglycemia. Compare with one abnormal items of OGTT parameters, patients with two or more abnormal items of OGTT parameters may suffer more serious glucose metabolic homeostasis disruptions and insulin sensitivity (20, 50). Our data presented patients with two or more abnormal items of OGTT parameters were of greater likelihood of adverse pregnancy outcomes, compared with patients with only one abnormal item of OGTT parameters. This indicated that more attention needs to be paid to strict management of hyperglycemia including diet control and exercise and pharmacological glucose-lowering is needed to prevent adverse pregnancy outcomes. However, overly strict glycemic control may often lead to SGA offspring, to severe diabetes. Our study showed no association between overweight or obesity, excessive gestational weight gain, two or more abnormal items of OGTT parameters, and SGA, and full- term birth weight. This may be because our participant received medical nutrition therapy after diagnosis, including interventions such as diet, exercise, and insulin therapy, these interventions result in good glycemic control so that they do not progress to severe uncontrolled diabetes. Previous studies have noted that GDM may result in SGA and preterm delivery and other neonatal complications (10, 20). Severe diabetes or overly strict severe diabetes or overly tight control may lead to SGA offspring. An observational study involved 2037 GDM women revealed average HbA1c levels in third trimester was a new risk factor for HDP in GDM women a new association between mean HbA1c levels and excessive weight gain and HDP has been established (47). Our data showed the strongest influence on HDP was exerted pre-pregnancy overweight or obesity, followed by excessive gestational weight gain. It may therefore be speculated that insulin resistance appears to be a key causative factor in HDP (51). Hyperglycaemia and various adipose tissue cytokines may be the mediators of systemic endothelial hypertensive vasculopathy (52).

There are complex relationships between overweight or obesity, gestational weight gain, maternal hyperglycemia, and adverse pregnancy outcomes, especially the interaction of them. Interaction analysis includes multiplicative interactions and additive interactions interaction analysis (53). Previous studies mainly focus on the association between overweight or obesity, and excessive gestational weight gain with adverse pregnancy outcomes, few studies evaluating the combined effect of pre-pregnancy overweight or obesity, excessive gestational weight gain and glucose tolerance status on pregnancy outcomes. An observational study examined the relative impact of a maternal factor on birth weight showed that pre-pregnancy BMI, and gestational weight gain was close with birth weight (54). A recently published literature found pre-pregnancy overweight or obesity increased the risk of macrosomia and LGA births independently and partly mediated by GDM (55). Black M H and colleagues conducted a retrospective study of 9,835women and revealed that pre-pregnancy overweight or obesity accounts for 23.3% of LAG in women with GDM and 21.6% in women without GDM (56). A large population-based study in Florida explored the adjusted population-attributable fraction of LGA as a result of the mutual effect of BMI, excessive gestational weight gain, and GDM and they discovered overweight and obesity, excessive gestational weight gain, and GDM all are associated with LGA, and excessive gestational weight gain has the greatest potential to reduce LGA risk (57). However, these studies mainly focus on neonatal birth weight-related outcomes, other adverse pregnancy outcomes, especially those related to the mother, were not addressed.

Our findings regarding fetal overgrowth are consistent with the above studies. We also found the association with adverse maternal outcomes. The mechanisms of pre-pregnancy overweight or obesity and excessive gestational weight gain are probably may be due to abnormal distribution of adipose tissue, which further contribute to impaired maternal metabolism, and an unhealthy intrauterine environment (58–60). Thus, pre-pregnancy overweight or obesity, excessive gestational weight gain, and GDM are inextricably linked. In our study, the high independent effects of excessive gestational weight gain on fetal overgrowth exceed that of pre-pregnancy overweight or obesity and two or more abnormal items of OGTT parameters, while the independent effect on HDP, cesarean section, and the composite outcome was the greatest for pre-pregnancy overweight or obesity. It is worth noting that the combination of each two and all have a greater impact than either one alone and the all combination was of the greatest. Previous studies also confirmed similar result (61–63).

Overweight or obese women may more likely to gain excessive weight, excessive gestational weight gain can aggravate insulin resistance, that is, increase the risk of developing GDM (64–69). However, it is important to mention that gestational weight gain as or below recommended among overweight or obese women didn’t lower the risk of macrosomia, LGA, HDP, cesarean section, and composite outcome, regardless of OGTT pathological values. What’s more, gestational weight gain as or below recommendations among underweight or normal women with two or more abnormal items of OGTT parameters was still associated with increased risk of macrosomia, LGA, and HDP. These findings contrast with those of previous studies which reported inadequate gestational weight gain in overweight or obese women with GDM lower risk of macrosomia (70), or do not effect on birth weight (64).

Our results presents the interplay between overweight or obesity, gestational weight gain, and GDM and their interaction with adverse pregnancy outcomes. Wherefore, pre-pregnancy overweight or obesity and gestational weight gain requires systematic monitoring and management before and during pregnancy. All women are encouraged to develop good dietary and living habits before pregnancy, especially among overweight or obese women. Observational study showed that adherence to a healthy lifestyle before pregnancy by maintaining a healthy weight, adhering to a healthy diet, regular exercise and avoiding smoking can prevent approximately 45% of GDM cases (71). And besides, diet and lifestyle changes early in pregnancy can also prevent GDM. A meta-analysis showed that lifestyle modification (diet, physical activity, or both) initiated before the 15^th^ week of gestation reduced the risk of GDM (72). Totally, the focus of GDM prevention efforts should be on the preconception phase or early pregnancy to achieve the desired reduction in the prevalence of GDM and the attendant pregnancy complications (48). After the diagnosis, the treatment of GDM is mainly to control the blood glucose level with target by dietary modification and promotion of physical activity to prevent fetal overgrowth and pregnancy complications (73–75). In addition to hyperglycemia, excessive gestational weight gain is also associated with fetal overgrowth and pregnancy complications in both healthy and GDM pregnant women (76, 77). Weight management services are recommended before conception to provide advice on weight optimization. In contrast with pre-pregnancy overweight or obesity, prevention of excessive gestational weight gain may be more feasible. Pregnancy offers a unique challenging time for women to be to informed of the long-term implications of overweight or obesity and excessive gestational weight gain on themselves and their fetal future health from obstetricians and midwives, help them take preventive and interventive measures to lower the risk of GDM and adverse pregnancy outcomes (78). Furthermore, women of reproductive age usually don’t know their blood glucose levels and thus miss out on preconception counseling and treatment. Our results suggested that excessive weight gain has the greatest impact on adverse pregnancy outcomes, followed by pre-pregnancy overweight or obesity. Thus, Whether pre-pregnancy overweight or obesity, or in those with two or more abnormalities in glucose tolerance during pregnancy, we recommend to monitor weight gain in each antenatal visit carefully and set weight-gain goals for patients to reduce GDM risk and improve adverse pregnancy outcomes. Little is known about the optimal gestational weight gain for women with GDM, and future research should focus on determining the appropriate weight gain for women with GDM. Pharmacological treatment should be initiated when glycaemia remains after 1-2 weeks of lifestyle intervention, of which insulin is the primary medical treatment also includes metformin or sulfonylurea (48).

There are some limitations to the study. First, due to retrospective design, confirmation of causal association is limited. Second, the category of BMI we used was Chinese standard, i.e., BMI≥24 is defined as overweight and ≥28 as obesity, which is different from the international definition in the literature and will have some limitations when comparing the results of others studies. Third, women with GDM will be treated after diagnosis and those interventions may underestimate the risk of adverse pregnancy outcomes. Although these limitations, our study is the first time to depicted the interactive association between overweight or obesity, excessive gestational weight gain, abnormal items of OGTT on adverse pregnancy outcomes. The second strength is that we adjusted for the potential mediating effect and considered results reliable.

In summary, our result demonstrates the independent multiplicative interaction but no additive interaction between overweight or obesity, gestational weight gain, and two or more abnormal items of OGTT parameters on adverse pregnancy outcomes among women with GDM. Every effort should be made for women to conceive with pre-pregnancy normal weight and reasonable gestational weight gain to reduce the risk of GDM and improve pregnancy outcomes.

## Data Availability Statement

The original contributions presented in the study are included in the article/supplementary material. Further inquiries can be directed to the corresponding authors.

## Ethics Statement

The studies involving human participants were reviewed and approved by Ethics committee of the Fujian Maternity and Children Hospital. Written informed consent for participation was not required for this study in accordance with the national legislation and the institutional requirements.

## Author Contributions

J-YY and JL contributed to study designed and critical revision of the manuscript. L-HL collected data, analyzed data, prepared and edited the manuscript. All authors contributed to the article and approved the submitted version.

## Conflict of Interest

The authors declare that the research was conducted in the absence of any commercial or financial relationships that could be construed as a potential conflict of interest.

## Publisher’s Note

All claims expressed in this article are solely those of the authors and do not necessarily represent those of their affiliated organizations, or those of the publisher, the editors and the reviewers. Any product that may be evaluated in this article, or claim that may be made by its manufacturer, is not guaranteed or endorsed by the publisher.
